# Analysis of HIV prevalence among pregnant women in Liangshan Prefecture, China, from 2009 to 2015

**DOI:** 10.1371/journal.pone.0183418

**Published:** 2017-09-07

**Authors:** Shujuan Yang, Chao Yang, Qiang Liao, Wenwen Zhai, Gang Yu, Lin Xiao, Qixing Wang, Yuhan Gong, Suhua Zhang, Yongna Yao, Ke Wang, Ju Wang, Shaochao Bian, Qian Liu

**Affiliations:** 1 Department of Health Related Social and Behavioral Science, West China School of Public Health, Sichuan University, Chengdu, China; 2 Department of Epidemiology and Statistics, School of Public Health, Southwest Medical University, Luzhou, China; 3 Liangshan Prefecture Center for Disease Control and Prevention, Xichang, China; Institut Pasteur of Shanghai Chinese Academy of Sciences, CHINA

## Abstract

**Background:**

Yi people make up about 50% of the population in Liangshan Prefecture, Sichuan Province, China, but accounted for 88.07% of new HIV cases in the prefecture from 2011 to 2013. This study evaluated HIV prevalence in pregnant women of Liangshan Prefecture using HIV sentinel surveillance (HSS) data over the period of 2009 to 2015.

**Methods:**

Xichang, Zhaojue County, and Butuo County were selected as HSS sites. We investigated the temporal trends in HIV prevalence in these areas, and the association between demographic and behavioral characteristics and risk of HIV infection.

**Results:**

Data on a total of 2797 pregnant women in Xichang and 3983 pregnant women in Zhaojue and Butuo was collected for the period 2009 to 2015. There was a fluctuating HIV prevalence among pregnant women of Xichang, with a rate of 0.75% in 2015 (*χ*^2^_trend_ = 2.27, P = 0.13). HIV prevalence among pregnant women of Zhaojue and Butuo was consistently high, varying between 3.4% (9/267, 2011) and 10.3% (82/796, 2012) over the period of 2010 to 2015 (*χ*^2^_trend_ = 0.12, P = 0.73). In Xichang, we found that Yi ethnicity (OR = 11.37, 95% CI = 2.92–44.25) and a husband who used drugs (OR = 32.13, 95% CI = 5.33–193.67) were significantly associated with HIV risk in pregnant women. For Zhaojue and Butuo, we observed that pregnant women had a higher risk of HIV infection when they were over 30 years old (OR = 1.72, 95% CI = 1.17–2.52), when they had a higher number of previous births, when their husbands had a history of migrating for work (OR = 1.56, 95% CI = 1.16–2.08), and when they had a history of other sexually transmitted infections (OR = 2.19, 95% CI = 1.16–2.08). Compared to those with a primary school education or below, pregnant women with a secondary or high school education level had a lower risk of HIV infection (OR = 0.28, 95% CI = 0.09–0.89).

**Conclusion:**

Our results indicate that there is a serious HIV epidemic among pregnant Yi women, especially for those with less education, more past births, or a husband with a history of out-migrating for work or STD infection.

## Introduction

Liangshan Prefecture reported its first HIV case in 1995, an injecting drug user (IDU). Since then, the incidence of HIV infection has greatly increased in this area, and Liangshan Prefecture now has one of the highest HIV prevalence rates in China [1]. As of December 2015, a total of 29,987 HIV/AIDS cases had been reported, and the HIV epidemic had spread throughout Liangshan’s 17 districts [2]. The region is located along drug trafficking routes from the "Golden Triangle" to west and central China [3–5]. Therefore, its high prevalence of HIV is due in part to injected drug use [6]. However, the HIV epidemic has gradually changed, with a shift in high-risk behaviors from IDU to heterosexual behaviors [2].

HIV sentinel surveillance (HSS) is an important strategy for monitoring the HIV epidemic. HSS in Liangshan Prefecture was first established in 1997 [7] for IDUs, and later expanded to many other populations, such as men who have sex with men (MSM), female sex workers, sexually transmitted disease (STD) clinic attendants, and pregnant women. Pregnant women are a key population to target for HIV prevention, as early diagnosis and interventions decrease the likelihood of mother to child transmission. HSS for pregnant women has been conducted in Liangshan Prefecture since 2009. In a previous study, we observed that HIV incidence in females increased between 2011 and 2013 in Liangshan Prefecture, and that females accounted for 41.63% of new HIV infections [1], suggesting that females in Liangshan Prefecture were increasingly at risk for infection.

The Yi people comprise the seventh largest ethnic group in China, with a population of about eight million people, many of whom live in Liangshan Prefecture [8]. Yi people in Liangshan Prefecture make up about 50% of its population, but accounted for 88.07% of new HIV infections from 2011 to 2013 [2]. We performed HSS for pregnant women at three sites in Liangshan Prefecture, including one site (Xichang) where people of Han ethnicity are the majority, and two sites (Zhaojue and Butuo) where Yi people are the majority. Understanding trends in the epidemic among pregnant women in Liangshan Prefecture is important in devising HIV prevention policies and strategies. In this study, we evaluated HIV prevalence among pregnant women of Liangshan Prefecture through HIV surveillance data over the period from 2009 to 2015.

## Materials and methods

### Ethics

All subjects voluntary participated in our study and signed informed consent forms before enrollment. This study was approved by the ethics committee of the STD and AIDS centers for disease control within the Chinese Center for Disease Control and Prevention (CDC), and the study was carried out in accordance with the Helsinki Declaration of 1964.

### HSS site selection

HSS sites in Liangshan Prefecture were selected to be representative of the prefecture’s HIV epidemic, based on data analysis by the Chinese CDC and the Sichuan CDC [9]. Xichang is the political and economic center of Liangshan Prefecture, and its population is majority Han, with Yi and other ethnic minorities accounting for 23.29% of the population, so it was selected to be representative of Han people in the prefecture. Zhaojue County (93.43% Yi population) and Butuo County (97.60% Yi population) both have majority-Yi populations who observe traditional Yi customs, and these two counties have the highest HIV infection rates in Liangshan prefecture [10, 11].

### Subjects

A cross-sectional study design was used. We enrolled pregnant women who received prenatal health care in the Liangshan Prefecture Maternal and Children’s Health Hospital, the Zhaojue County Maternal and Children’s Health Hospital, and the Butuo County Maternal and Children’s Health Hospital between April and June over the period of 2009 to 2015. HSS was performed in accordance with the "Implementation Plan of the National HIV Sentinel Surveillance" introduced by the Chinese CDC in 2009. The inclusion criteria were pregnant women who originally registered for childbirth in HSS, and pregnant women who registered in other hospitals but first received perinatal health care in HSS. The pregnant women were monitored until giving birth. Women who came to HSS for pregnancy termination were excluded. According to protocols from the "Implementation Plan of the National HIV Sentinel Surveillance,” 400 eligible pregnant women were enrolled at each sentinel site every year. If the sample size did not reach to 400 by the end of June, the sentinel surveillance period would extend to the end of August at the latest.

A total of 6812 pregnant women were enrolled from 2009 to 2015, 32 of which were missing HIV test results and were dropped. Finally, 2797 valid cases in Xichang and 3983 in Zhaojue and Butuo were enrolled.

### Questionnaire

The questionnaire was accompanied by an introduction of the study purpose. It was designed by the STD and AIDS centers for disease control of the Chinese CDC (S1 and S2 Tables).

The questionnaire was two pages long, and included demographic characteristics, HIV-related prevention knowledge, and behavioral characteristics of both the pregnant women and their husbands. The demographic characteristics collected were age, ethnicity, and education level. HIV-related prevention knowledge was assessed via eight items, each expressed with a two-point Likert scale of 0 (no) to 1 (yes). A total score above five was considered to indicate awareness of HIV-related prevention knowledge; otherwise, the patient was considered unaware of effective HIV prevention principles. The behavioral characteristics collected for pregnant women and their husbands included history of migrating for work, drug use, and sexually transmitted diseases (STDs); sexual history with other men and women; unprotected sexual activity within marriage; and commercial sexual activity in the past six months.

The questionnaire was administered to all participants via a face-to-face interview conducted by investigators who received uniform training. Subjects were informed the purpose of our study but not of the research hypothesis.

### HIV testing

Before the interview, each subject provided 3–5 ml of venous blood for HIV testing. Specimens were tested for HIV at the Liangshan Prefecture CDC. Enzyme-linked immunosorbent assay (ELISA) was used to screen for HIV antibodies. Negative ELISA results indicated no HIV infection, while those with positive results were re-tested for HIV antibodies by ELISA with either different reagents or reagents from a different manufacturer. If the second test for HIV was positive, the serum was confirmed as positive for HIV antibody. If negative, the serum was regarded as negative for HIV antibodies.

### Statistical analysis

Categorical variables were reported as percentages and frequencies, and continuous variables were presented as means ± standard deviations. Categorical variables were compared between the two investigated groups with the Pearson chi-square (χ^2^) or Fisher’s exact test, while comparison for continuous variables was done by the Student’s t-test. A multiple forward stepwise logistic regression model was used to evaluate the relationship between demographic and behavioral characteristics and risk of HIV infection overall over the period from 2009 to 2015. The results were expressed as odds ratios (ORs) and 95% confidence intervals (CIs). Statistical analyses were carried out using SPSS version 17.0 for Windows (SPSS, Inc., Chicago, IL, USA). P < 0.05 was considered to indicate statistically significant difference, and all P values were two-tailed.

## Results

A total of 2797 pregnant women in Xichang and 3983 pregnant women in Zhaojue and Butuo were enrolled from 2009 to 2015, and all subjects received HIV testing (Table 1). In Xichang, Yi ethnicity, lower education level, older age, more past births, and a husband with a history of drug use were associated with a greater risk for HIV infection. In Zhaojue and Butuo, factors associated with HIV risk were lower education level, older age, more past births, a history of out-migrating for work, and a husband’s history of out-migrating for work, drug use, and STDs.

**Table 1:** Demographic characteristics, awareness of HIV-related prevention knowledge, and behavioral characteristics of included subjects.

Variables	Xichang N = 2797				*χ*^2^	P	Zhaojue and Butuo N = 3983				*χ*^2^	P
	HIV-positive	%	HIV-negative	%			HIV-positive	%	HIV-negative	%		
	N = 11		2786				N = 277		N = 3706			
**Ethnicity**												
Yi	8	1.79	439	98.21	26.48	<0.001	276	7.03	3648	92.97	-	0.125
Han or other	3	0.13	2347	99.87			1	1.69	58	98.31		
**Education level**												
Primary school or below	7	1.79	383	98.21	-	<0.001	272	7.23	3492	92.77	-	0.011
Secondary or high school	3	0.18	1666	99.82	12.77^a^	<0.001^a^	3	1.94	152	98.06	6.356^a^	0.012^a^
Junior college or above	1	0.14	737	99.86			2	3.13	62	96.88		
**Age, yrs**												
Mean age	30.73±6.640		26.54±4.82		-2.87	0.004	30.36±6.80		29.61±7.35		-1.65	0.07
<25	2	0.19	1039	99.81	-	0.189	55	5.11	1022	94.89	9.2	0.01
25–30	4	0.36	1120	99.64	3.36^a^	0.08^a^	75	6.91	1010	93.09	9.43^a^	0.01^a^
≥30	5	0.79	627	99.21			147	8.07	1674	91.93		
**Number of previous births**												
0	5	0.27	1818	99.73	-	0.013b	40	4.80	794	95.20	14.64	0.002
1	3	0.39	774	99.61	8.00^a^	0.01^a^	83	7.66	1001	92.34	1.09^a^	0.30^a^
2	1	0.64	156	99.36			97	8.85	999	91.15		
3 or above	2	5.00	38	95.00			57	5.88	912	94.12		
**History of out-migrating for work**												
No	9	0.45	2003	99.55	-	0.738	238	6.67	3330	93.33	4.27	0.04
Yes	2	0.25	783	99.75			39	9.40	376	90.60		
**Husband’s history of out-migrating for work**												
No	8	0.41	1932	99.59	-	1	211	6.41	3083	93.59	8.87	0.003
Yes	3	0.35	854	99.65			66	9.58	623	90.42		
**Awareness of HIV-related prevention knowledge**												
No	4	0.49	806	99.51	-	0.53	70	7.83	824	92.17	1.37	0.24
Yes	7	0.35	1980	99.65			207	6.70	2882	93.30		
**Drug use**												
No	11	0.39	2782	99.61	-	1	277	6.99	3684	93.01	-	0.79
Non-injection drug use	0	0.00	2	100.00			0	0.00	14	100.00		
IDU	0	0.00	2	100.00			0	0.00	8	100.00		
**Husband with drug use**												
No	9	0.32	2772	99.68	-	0.002	263	6.77	3619	93.23	7.64	0.01
Yes	2	12.50	14	87.50			14	13.86	87	86.14		
**Sexual history with men other than husband during marriage**												
No	10	0.36	2760	99.64	-	0.1	263	6.83	3586	93.17	2.62	0.11
Yes	1	3.70	26	96.30			14	10.45	120	89.55		
**History of STD**												
No	13	0.40	2773	99.60	0.05	0.82	22	6.88	2784	93.12	5.62	0.18
Yes	0	0.00	11	100.00			5	18.52	3684	81.48		
**History of STD of husband**												
No	10	0.36	2769	99.64	-	0.07	261	6.73	3618	93.27	11.73	0.001
Yes	1	5.56	17	94.44			16	15.38	88	84.62		

^a^: Trend chi-square test.

**Fig 1:**
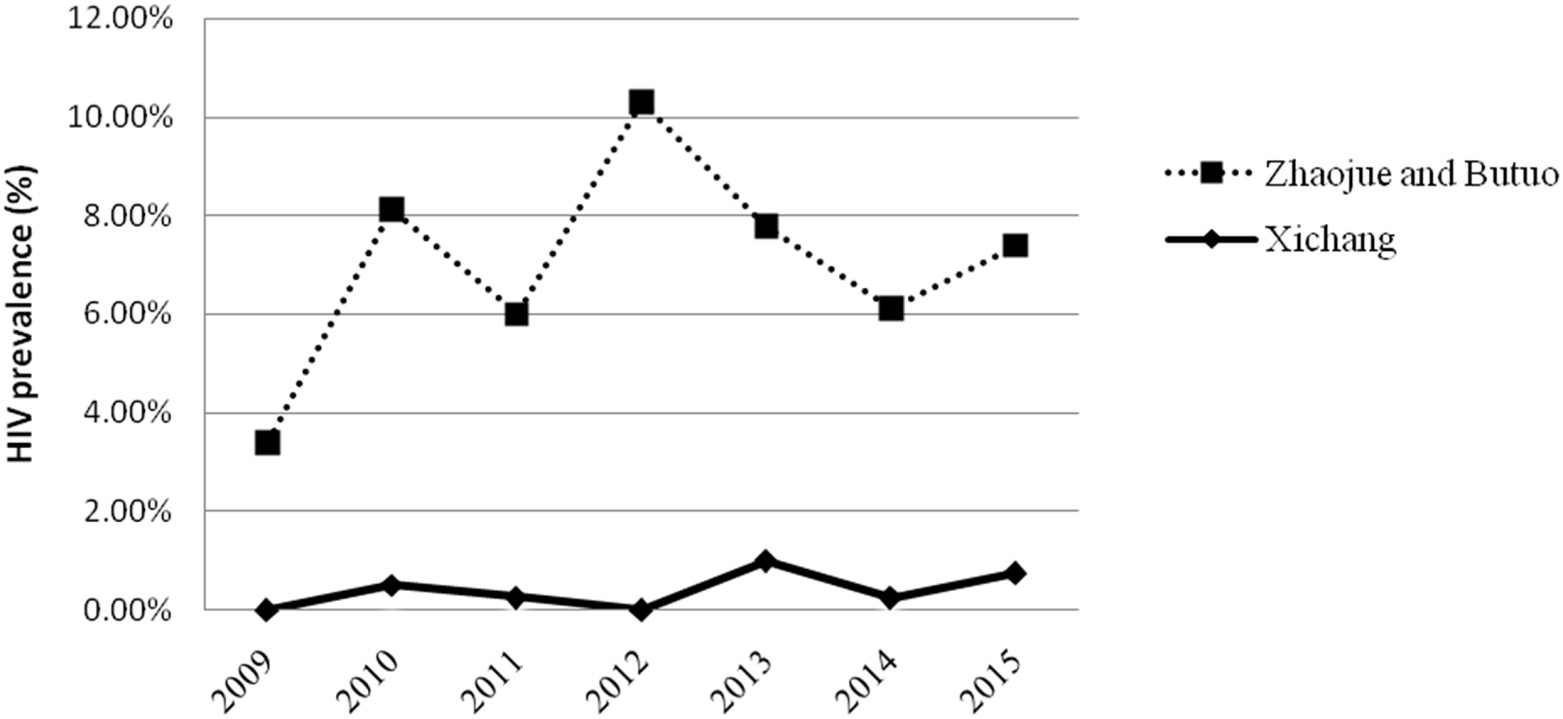
HIV prevalence in pregnant women.

The HIV prevalence in the pregnant women of Xichang was 0% (0/398) in 2009 and 2012 and varied by year, peaking at 1.0% (4/400) in 2013. The temporal variation was not significant (*χ*^2^_trend_ = 2.27, P = 0.13) (Fig 1).

In Zhaojue and Butuo, HIV prevalence in pregnant women was relatively low in 2009 (3.4%, 9/267). It reached a peak of 10.3% (82/796) in 2012, and was 6.6% (53/800) in 2015. It remained high and varied between 5.7% (35/610, 2011) and 10.3% (82/796, 2012) over the period of 2010 and 2015. The temporal variation in HIV prevalence for these areas was also not significant (*χ*^2^_trend_ = 0.12, P = 0.73) (Fig 1).

In Xichang, Yi ethnicity (OR = 11.37, 95% CI = 2.92–44.25) and having a husband with drug use (OR = 32.13, 95% CI = 5.33–193.67) were significantly associated with HIV risk in pregnant women (Table 2).

**Table 2:** Multivariate logistic regression analysis of factors associated with HIV positivity in pregnant women of Xichang.

Variable	P	OR	95% CI
**Ethnicity**			
Han or other ethnicity		1.0 (Reference)	
Yi ethnicity	0.001	11.37	2.92–44.25
**Husband with drug use**			
No		1.0 (Reference)	
Yes	<0.001	32.13	5.33–193.67

OR: odds ratio; CI: confidence interval.

In Zhaojue and Butuo, significantly higher HIV risk was observed in pregnant women with age over 30 years (OR = 1.72, 95% CI = 1.17–2.52), more previous births (1 birth vs. 0 births: OR = 1.58, 95% CI = 1.07–2.33; 2 vs. 0: OR = 1.81, 95% CI = 1.23–2.65), and a husband with a history of out-migrating for work (OR = 1.56, 95% CI = 1.16–2.08) or of STD infection (OR = 2.19, 95% CI = 1.16–2.08). Compared to pregnant women with a primary school education or below, those with a secondary or high school education had a lower risk of HIV infection (OR = 0.28, 95% CI = 0.09–0.89) (Table 3).

**Table 3:** Multivariate logistic regression analysis of factors associated with HIV positivity in pregnant women of Zhaojue and Butuo.

Variable	P	OR	95% CI
**Age, yrs**			
<25		1.0 (Reference)	
25–30	0.20	1.29	0.88–1.89
≥30	0.005	1.72	1.17–2.52
**Education level**			
Primary school or below		1.0 (Reference)	
Secondary or high school	0.03	0.28	0.09–0.89
Junior college or above	0.32	0.49	0.12–2.01
**Husband’s history of out-migrating for work**			
No		1.0 (Reference)	
Yes	0.005	1.52	1.14–2.03
**Husband's history of STD infection**			
No		1.0 (Reference)	
Yes	0.002	2.38	1.37–4.13
**Number of previous births**			
0		1.0 (Reference)	
1	0.018	1.58	1.07–2.33
2	0.001	1.81	1.23–2.65
≥3	0.322	1.15	0.76–1.75

OR: odds ratio; CI: confidence interval.

## Discussion

This study investigated trends in HIV infection among pregnant women of Liangshan Prefecture over a seven-year period. HIV prevalence in this population remained low and stable in the city of Xichang over the study period, while it was higher and stable in Zhaojue and Butuo counties. The HIV prevalence rates of all three sites were significantly higher than for China as a whole (0.058%) [12] or Sichuan Province (0.17%) [13], suggesting that the HIV epidemic among pregnant women is serious in Liangshan Prefecture, especially in Zhaojue and Butuo.

Our study found that HIV prevalence in pregnant women of Zhaojue and Butuo varied between 3.4% and 10.3%, while it was at 0.75% in Xichang in 2015. In 2002, Liu et al. conducted a study in Xichang with 824 pregnant women and did not find any HIV-positive cases [14]. However, HIV prevalence had increased to 0.75 in Xichang and 6.6% in Zhaojue and Butuo in 2015. HIV prevalence in Zhaojue and Butuo was significantly higher than that in the four Chinese provinces with the highest HIV infection rates. Zhao et al. conducted a study in Chuxiong Yi Autonomous Prefecture of Yunnan Province with 2887 pregnant women, and found an HIV prevalence of 0.10–0.50% [15]. Chen et al. performed a study with 28,078 pregnant women in Guangxi Province, and reported an HIV prevalence of 0.10% [16]. In Henan Province, Sun et al. conducted a study with 763,514 pregnant women; their HIV prevalence varied between 0.06% and 0.54% over the period of 2001 to 2006 [17]. Lu et al. performed a study in Xinjiang Province with 4235 pregnant women, and reported a prevalence rate of 0.26% [18]. In our study, the HIV prevalence in Xichang (where most subjects were Han) was slightly higher than that in other provinces of China, such as Jiangsu (HIV prevalence = 0.19%) [19] and Guizhou (HIV prevalence = 0.27%) provinces [20].HIV prevalence among pregnant women in Butuo and Zhaojue was significantly higher than that in Xichang in the current study. Several reasons contribute to the high prevalence in Butuo and Zhaojue, including a long history of opium cultivation, sharing traditions, economic and education levels, and cultural sexual traditions.

IDU is reportedly the primary mode of infection for HIV in Sichuan Province, and more than 50% of the province’s HIV infections occur in Liangshan Prefecture [21]. Liangshan Prefecture is located along drug trafficking routes from the "Golden Triangle" to west and central China [13,14], and has a long history of opium cultivation and opium/heroin trade [22]. Moreover, sharing is an integral part of traditional Yi culture, leading drug users to share both drugs and needles. In our study, we observed that having a husband who used drugs was associated with a higher risk for HIV infection among pregnant women in Zhaojue and Butuo, suggesting that unprotected sexual behavior within marriage may spread the infection from IDUs to their spouses.

In our previous study, we observed that 84.20%-94.59% of new HIV infections in Liangshan Prefecture between 2011 and 2013 were among Yi people, and approximately 60% occurred among people with primary school or less education [2]. In this study, 98.19% (272/277) of the HIV-infected pregnant women were Yi people and had primary school or less education, which is in line with our previous findings. Yi people generally live in high-altitude, cold mountain areas, and economic poverty and low education levels are very common in this ethnic group. Children must often travel very far over mountainous terrain from home to school, and thus children who fall below the expected grade level and high dropout rates are common in this population. In addition, the Yi language is quite different from Mandarin Chinese, and many Yi students can't understand the contents of their classes due to the language barrier; thus, "difficulty in understanding and learning, and poor grades" is the main reason students give for dropping out of school [23]. A recent report indicated that the enrollment rate of rural children was about 64.8% in Yi people of Liangshan Prefecture, and the rate of illiteracy or semi-illiteracy among young people was as high as 70% [24]. In addition, lower education levels likely correspond with lack of knowledge about STDs and AIDS. People living in poverty may not be able to afford condoms. These factors could be expected to increase behaviors that put people at high risk for HIV infection. Language differences are another possible barrier to preventing high-risk behaviors: many less educated Yi people may not understand available AIDS/HIV prevention interventions and health education, which are mainly conducted by Han people. Therefore, poverty and lack of education are some probable reasons for the high HIV prevalence in this population.

The cultural sexual traditions of Yi people also contribute to HIV infection risk [25–27]. For example, Yi people tend to engage in more frequent casual sexual behavior than the Chinese population as a whole [26, 28]. Yang et al. found that more than 30% of Yi people hold permissive attitudes toward premarital sexual behavior and extramarital sexual behavior before and after cohabitating, and 66.7% of the investigated subjects had multiple sexual partners in the year leading up to the survey [28]. Multiple sexual partners and more frequent casual sexual behavior may promote HIV transmission from IDUs to the general population in Liangshan Prefecture.

Another HIV risk factor identified in our study was number of previous births. Traditionally, Yi people prefer sons to daughters and have large families. Most Yi families have many children even when they live in deep poverty [24]. In some families, HIV-infected husbands do not use a condom during intercourse within marriage because they wish to have a son, a practice that can spread HIV infection within the family [25]. Therefore, cultural preferences for sons and large families can contribute to the spread of infection in this ethnic group.

We observed that a husband's history of out-migrating for work and of STDs were risk factors for HIV infection (OR = 1.56, 95% CI = 1.16–2.08). Male farm migrants may have a greater likelihood of engaging in extramarital sexual behavior, casual sexual behaviors, and commercial sexual behavior, because they are separated from their spouses for long periods. Our previous study found that outmigration for work is a risk factor for HIV infection among unmarried youths in Liangshan Prefecture [26]. Wu et al. conducted a study in migrant farmer workers of Sichuan Province, and reported that 25% of the subjects had engaged in high risk sexual behavior such as extramarital sex, multiple sexual partners, casual sex, and commercial sex [29]. Another investigation indicated that male migrants with low incomes and less education were more likely than the population as a whole to take on temporary sexual partners, and of those who did, 76.2% never or only occasionally used condoms [30]. These studies indicate that male migrant workers are more likely to have unprotected sex with temporary partners; they may then transmit HIV and other STDs to their wives due to unprotected sexual behavior within marriage.

Three limitations should be considered in this study. One is that risk factors for HIV were self-reported. Because sexual history and drug use are sensitive subjects, people may have concealed the truth about their sexual history with men other than their husband during marriage or their drug use. It is also possible some subjects were not aware of their husbands’ sexual history or drug use. Therefore, an anonymous questionnaire could be used in further studies. Second, we did not observe that the Yi ethnicity was associated with HIV risk in Zhaojue and Butuo, perhaps due to the small number of Han pregnant women at these two sites. Moreover, the sample sizes of HIV-positive cases were small in Xichang and in some subgroup analyses, reducing the statistical power to find differences between groups. Therefore, prospective studies with large sample sizes are needed to confirm our findings.

## Conclusions

Our results suggest that the HIV epidemic in Liangshan Prefecture is very serious among pregnant Yi women, especially for those with less education, more past births, or a husband with a history of out-migrating for work or STD infection. It is important to promote health education that uses the local language and culture to target young adults who are sexually active. Multi-pronged interventions should be used to target unprotected sexual behaviors in Yi people, and HIV testing and treatment for HIV/AIDS patients should be widely available in this area.

## Supporting information

S1 Table The survey questionnaire in English. (PDF)

S2 Table The survey questionnaire in Chinese. (PDF)
