# Supplementary material for: Analysis of HIV prevalence among pregnant women in Liangshan Prefecture, China, from 2009 to 2015
Source: PLoS One. 2017 Sep 7;12(9):e0183418. doi: 10.1371/journal.pone.0183418 (PMC5589085; doi:10.1371/journal.pone.0183418)
Supplement: S1 Table — (PDF) [file pone.0183418.s001.pdf]

materna questionnaire

Health Questionnaire（XII）

A01 Monitoring site___________province（municipality）________city __________county
__________________________________ unit（Monitoring object specific collecting unit）

A02 The sentinel type PRG

A03 The sentinel local administrative divisions gb code □□□□□□

A04 Questionnaire number □□□（001—999）

A05 Survey date □□□□ year □□ month □□ day

Hi, my name is..., from.... For the health of you and your fetus, we need to know conditions of your knowledge and behavior. The survey is not registered, We will keep secret for your answer, Hope you to provide your real situation.The investigation will take about10 minutes.At the end of the survey I can provide some help for you (for example, you can consult some health problems, I will try to answer).I hope you will support our wor.Thank you!

Ask respondents: Can you tell me whether you have recently participated in the survey? If answered "yes" to the end of the visit.

B01 Year of birth _____________Year

B02 Marital status ①Unmarried ②Married ③Cohabitation ④Divorced or widowed

B03 Census register ①This province ②Other provinces(Please specify _______province）
③foreign（Please specify _______ country）（Skip to B05）

B04 Nationality

B05 Degree of education ①Illiteracy ②Primary grades ③Junior high school ④High school or technical secondary school ⑤College graduate or above

B06 The pregnancy gestational age _____________week

B07 pregnant number _____________times

B08 Reproductive number ____________ times

B09 Have you been to other places working or doing business? ① Yes ② No

B10 Have your husband been to other places working or doing business? ① Yes ② No

C01 Can a person infected with HIV be seen on the surface?① Yes ② No ③I don't know

C02 Can mosquito bites spread HIV/AIDS? ① Yes ② No ③I don't know

C03 With HIV infected people or patients eating together, will we be infected with AIDS?
① Yes ② No ③I don't know

C04 Does the transmission of blood with HIV virus cause AIDS? ①Yes ②No ③I don't know

C05 Is it possible to get AIDS sharing syringes with people infected with HIV?
①Yes ②No ③I don't know

C06 Is baby born to women infected with HIVpossible to get AIDS?①Yes ②No ③I don't know

C07 Can condom use reduce the spread of AIDS? ①Yes ②No ③I don't know

C08 Can we reduce the spread of AIDS if our sexual activity is associated with only one sex partner ①Yes ②No ③I don't know

D01 Do your husband take drugs? ①Yes ②No

D02 Do you take drugs? ①Yes ②No（Skip to E01）③Refuse to answer

D03 Have you ever injected drugs? ①Yes ②No（Skip to E01）③Refuse to answer

D04 Have you ever shared needle with others ? ①Yes ②No（Skip to E01）③Refuse to answer

E01 Do you have any other sexual partners than the husband?

①Yes ②No（Skip to E01）③Refuse to answer

F01 Have your husband had HIV/AIDS/STD? ① HIV/AIDS ②STD ③HIV/AIDS/STD

④No ⑤I don't know ⑥Refuse to answer

F02 Have you had HIV/AIDS/STD ? ①Yes ②No（End）

F03 In recent year, What kind of sexually transmitted disease have you ever been diagnosed?

①gonorrhoea ②syphilis ③Genital tract chlamydia trachomatis infection ④condyloma

⑤herpes progenitalis ⑥other（Please specify ________） ⑦Refuse to answer

---

Now,investigation is end, thank you for your cooperation.To understand your healt, We need to take your blood for syphilis and HIV testing.

T01 Have been taken blood for this investigation ①Yes（Skip to T03） ②No

T02 Why have not been taken blood for this investigation

①previously detect HIV antibody positive ②Refuse to be taken blood

T03 The HIV antibody test results

For the first time at the beginning of ELISA screen ①positive ②negative（Skip to T04）

The second reinspect ELISA ①positive ②negative

Validation test ①positive ②negative ③suspicious ④Did not detect

T04 Treponema pallidum antibody test results ①positive ②negative

Investigator ________________ supervisor
