# Supplementary material for: Analysis of HIV prevalence among pregnant women in Liangshan Prefecture, China, from 2009 to 2015
Source: PLoS One. 2017 Sep 7;12(9):e0183418. doi: 10.1371/journal.pone.0183418 (PMC5589085; doi:10.1371/journal.pone.0183418)
Supplement: S2 Table — (PDF) [file pone.0183418.s002.pdf]

健康调查问卷（XII）

A01 监测地点 ________省（自治区、直辖市）________市________县（市、区）
________单位（监测对象具体征集单位）

A02 哨点类型 PRG

A03 哨点所在地行政区划国标码 □□□□□□

A04 问卷编号 □□□（001—999）

A05 调查日期 □□□□年□□月□□日

你好，我叫……，来自……。为了你和胎儿的健康，我们需要了解你的行为和知识状况。本次调查不记名，我们会对你的回答保密，希望你提供你的真实情况。调查大约会占用你 10 分钟时间，调查结束时我可以为你提供一些帮助（例如你可以咨询一些健康方面的问题，我会尽量解答）。希望你支持我们的工作。谢谢！

询问调查对象：请问你最近是否参加过此项调查？若回答“是”则结束此次访问。

B01 出生年 ________年

B02 婚姻状况 ①未婚 ②在婚 ③同居 ④离异或丧偶

B03 户籍所在地 ①本省 ②外省（请注明________省） ③外籍（请注明________国）（跳至 B05）

B04 民族 ________族

B05 文化程度 ①文盲 ②小学 ③初中 ④高中或中专 ⑤大专及以上

B06 本次怀孕孕周 ________周

B07 怀孕次数 ________次

B08 生育次数 ________次

B09 本人是否去过外地打工或经商？ ① 是 ② 否

B10 您丈夫是否去过外地打工或经商？ ① 是 ② 否

C01 一个感染了艾滋病病毒的人能从外表上看出来吗？ ①能 ②不能 ③不知道

C02 蚊虫叮咬会传播艾滋病吗？ ①会 ②不会 ③不知道

C03 与艾滋病病毒感染者或病人一起吃饭会感染艾滋病吗？ ①会 ②不会 ③不知道

C04 输入带有艾滋病病毒的血液会得艾滋病吗？ ①会 ②不会 ③不知道

C05 与艾滋病病毒感染者共用注射器有可能得艾滋病吗？ ①可能 ②不可能 ③不知道

C06 感染艾滋病病毒的妇女生下的小孩有可能得艾滋病吗？ ①可能 ②不可能 ③不知道

C07 正确使用安全套可以减少艾滋病的传播吗？ ①可以 ②不可以 ③不知道

C08 只与一个性伴发生性行为可以减少艾滋病的传播吗？ ①可以 ②不可以 ③不知道

D01 你丈夫是否吸毒？ ①是 ②否

D02 你吸毒吗？ ①是 ②否（跳至 E01） ③拒答

D03 你注射过毒品吗？ ①是 ②否（跳至 E01） ③拒答

D04 你与别人共用过针具吗？ ①是 ②否 ③拒答

E01 你有丈夫以外的其他性伙伴吗？ ①是 ②否 ③拒答

F01 你丈夫是否患过艾滋病/性病？　　① 艾滋病　②性病　③艾滋病/性病 ④否　⑤不知道　⑥拒答

F02 你是否患过性病？　　①是　②否（结束）

F03 最近一年，你曾被诊断患过何种性病？

①淋病　②梅毒　③生殖道沙眼衣原体感染 ④尖锐湿疣 ⑤生殖器疱疹　⑥其它（请注明________）⑦拒答

调查到此结束，谢谢你的合作。为了解你的健康状况，我们需要对你采血，进行梅毒和艾滋病检测。

T01 本次调查是否采血		①是（跳至 T03）		②否	
T02 本次调查未采血原因		①既往检测 HIV 抗体阳性		②拒绝采血	
T03 HIV 抗体检测结果	第一次 ELISA 初筛	①阳性	②阴性（跳至 T04）		
	第二次 ELISA 复检	①阳性	②阴性		
	确认试验	①阳性	②阴性	③可疑	④未检测
T04 梅毒检测结果		①阳性	②阴性		
T05 丙肝检测结果：	第一次 ELISA 初筛	①阳性	②阴性（结束）		
	第二次 ELISA 复检	①阳性	②阴性		

调查员签字 ________________　　　　督导员签字________________
